# Effects of a ‘Rebuilding Myself’ intervention on enhancing the adaptability of cancer patients to return to work: a randomized controlled trial

**DOI:** 10.1186/s12885-024-12305-7

**Published:** 2024-05-13

**Authors:** Yujie Guo, Huiwen Xie, Lingyan Ding, Yue Shi, Pingping Han

**Affiliations:** School of Nursing and Rehabilitation, Nantong University, Nantong, 226001 Jiangsu Province China

**Keywords:** Cancer, Return to work, Adaptability, Nursing intervention, Randomized controlled trial

## Abstract

**Objectives:**

To explore the effects of a ‘Rebuilding Myself’ intervention on enhancing the adaptability of cancer patients to return to work.

**Methods:**

A single-center, single-blind, randomized controlled trial design was used. Eligible patients who were receiving routine hospital treatment were recruited from the university-affiliated hospital in our city. Patients in the control group only received usual care, while patients in the intervention group received additional ‘Rebuilding Myself’ intervention. Adaptability to return to work, self-efficacy of returning to work, mental resilience, quality of life and work ability were measured at baseline, the 6th and 12th of the intervention. The general estimation equations were used to compare the overall changes of each outcome index between the two groups at different time points. Considering that there may be patient shedding and rejection, Per-Protocol and Intention-to-Treat analysis were used to analyze the data in this study.

**Results:**

There were statistically significant differences between the two groups of patients in the cancer patients’ adaptability to return to work, self-efficacy to return to work, mental resilience, work abilities, the physical, emotional, cognitive function, fatigue, insomnia and overall health status dimensions of quality of life (*P* < 0.05). And no significant difference was found in other dimensions (*P* > 0.05). The group effect, time effect, and interaction effect of patients’ return to work adaptability and return to work self-efficacy were statistically significant in both groups (*P* < 0.05). Mental resilience, working ability, and quality of life had obvious time effect and interaction effect (*P* < 0.05).

**Conclusion:**

This intervention could improve cancer patients’ adaptability to return to work, self-efficacy to return to work, mental resilience, work abilities and quality of life. And it can be further expanded to improve the adaptability of patients to return to work, then to help patients achieve comprehensive rehabilitation.

**Implications for cancer survivors:**

The application of ‘Rebuilding Myself’ interventions can effectively improve the adaptability of cancer patients returning to work.

**Trial registration:**

This study was registered at the Chinese Clinical Trial Registry (Registration number: ChiCTR2200057943) on 23 March, 2022.

## Introduction

Cancer is the second leading cause of death worldwide [1]. As reported by the World Health Organization, the International Agency for Research on Cancer predicts that there will be 28.4 million new cases of cancer worldwide by 2040, an increase of 47% compared to 2020 [2]. Recently, the incidence of cancer in China has been steadily increasing [3], and what’s more, virtually all cancer types are becoming more common in younger age groups [4]. According to the Chinese Cancer Control Center [5], more than 1.4 million new cancer survivors are born every year. Working age cancer survivors have grown into a sizable population that should not be overlooked in the health management of residents. Clinical care and treatment options for cancer patients are constantly improving, contributing to an increase in the 5-year survival rate. Among cancer survivors, there will be increased problems returning to work after cancer treatment [6]. Long-term disease diagnoses, radiation, and chemotherapy, on the other hand, altered their career trajectories and had a significant impact on their ability to work [7]. One study found that cancer patients are 1.37 times more likely to be unemployed than healthy people [8].

For cancer patients, but also for their families and society, returning to work is an essential step in their rehabilitation [9]. This is defined as the resumption of paid work, full-time or part-time, including self-employment, in a regular or modified capacity for an average number of working hours per week [10]. Getting back to work is a critical factor for cancer patients to participate in life [11]. Returning to work can help the individual and family to reduce financial burden [12], improve social communication, make them feel appreciated, and increase self-esteem [13], all of which can improve quality of life [14]. Studies have shown that patients who have returned to work suffer less anxiety, depression and fear than patients who have not returned to work [15]. Returning cancer survivors to the workplace can help society by increasing the labor force, reducing social responsibility, and promoting harmonious social growth. Consequently, returning to work has a significant impact on both patients and society.

Nevertheless, a recent survey indicates that cancer patients in China have limited prospects of returning to work [8, 16, 17]. Although cancer patients were eager to return to work, they have had difficulty adjusting to the intensity of returning to work due to a number of medical, psychological and social factors. These include self-stigmatization of the disease, fear of cancer recurrence, reluctance to work, a lack of support from family, workplace and peers, and a lack of social roles [18–20]. Zhong ZJ et al [21] defined return-to-work adaptability as the ability to mobilize coping resources and adapt to the environment when individuals return to their original job or find new jobs and take on corresponding tasks after leaving their jobs due to injury or illness. One study showed [19] that 41.3% of cancer patients were poorly adapted to returning to work and that those who were employed, had positive coping, had high self-efficacy and family closeness, and had low illness stigma had better adaptability when returning to work. At present, there is still a lack of research at home and abroad that would address the above issues and improve patients ability to return to work after treatment.

In the early stages, our research group developed the ‘Adaptation Experience and Coping Resource Model for Cancer Patients to Return to Work’ [20]. In this model, the adaptive capacity of cancer patients to return to work represents a process of rebuilding themselves through the use of superior resources. There were three phases of adaptation: rehabilitation, rebuilding self-efficacy, adjusting and planning. In this category, rebuilding oneself was at the core. We have developed an assessment scale [22] based on this research and explored the factors that influence cancer patients’ adaptability to returning to work [19]. What’s more, we have also constructed an intervention protocol to improve the adaptability of returning to work for cancer patients before [23], and carried out a feasibility and pilot study. Therefore, the aim of this study was to investigate the effectiveness of a randomized controlled trial of the ‘Rebuilding Myself’ intervention to improve cancer patients’ adaptability when returning to work, to help them balance their physical, psychological, and social health, and finally to help them fully recover from cancer.

## Methods

### Study design

This research was carried out between January 2022 and December 2022. A single-blind, randomized, controlled trial was conducted at a single center with two parallel groups, and the paper was prepared in accordance with the Consolidated Standards for Reporting Trials (CONSORT 2010) [24]. Based on the methodology of the formulation and evaluation of a complex intervention plan [25], we followed the steps of formative research protocol, feasibility study, pilot study, and randomized controlled trial study to carry out the project, and this study was the last step of this project.

### Eligibility criteria

#### Inclusion criteria

This study enrolled patients whose conditions were as follows: (1) were diagnosed with a malignant tumor; (2) were receiving routine hospital treatment, and the disease had no progress or distant metastasis; (3) worked prior to treatment and had not yet returned to work; (4) were aged between 18 and 59 years old; (5) had a certain ability to read, write and understand; (6) were aware of their illness.

#### Exclusion criteria

Reasons for exclusion included: (1) cognitive disorder or mental disorder, accompanied by other serious complications; (2) participants in other related research projects at the same time.

#### Elimination criterion

Patients with disease recurrence, metastasis and deterioration were excluded during the intervention.

### Setting and participants

This study adopted the recruitment method of face-to-face and being recommended by medical staff. At the Affiliated Hospital of Nantong University, a convenient sampling method was used to recruit cancer patients.

### Measures

The intervention was evaluated using self-completion questionnaires at baseline, during the 6th week, and after the 12th week. Neither the intervention nor the analysis of the data were carried out by the investigator who collected the data.

#### Baseline

The general demographic questionnaire was developed by our research team in order to assess the general demographic characteristics of cancer patients as well as the disease characteristics of cancer patients. Age, gender, marital status, education level, place of residence, religious beliefs, medical insurance methods, disease types, disease stages, and treatment methods were all considered.

#### Primary effectiveness outcome

The primary effectiveness evaluation index was cancer patients’ adaptability to returning to work [20]. As a result of our research group’s efforts, this scale has proven to be reliable and valid. The Cronbach’s α coefficient of 0.973 was obtained for the entire scale. This scale consisted of 24 items divided into three dimensions: focus on rehabilitation (6 items), rebuild self-efficacy (9 items), and adjust and plan (9 items). Using a 5-point Likert scale, ‘strongly agree’ = ‘5’, ‘strongly disagree’ = ‘0’. A higher total score indicates a greater level of adaptability to return to work on the part of the patient.

#### Secondary effectiveness outcomes

The secondary effectiveness evaluation indexes were self-efficacy of returning to work, mental resilience, quality of life, and work ability.

Patients’ self-efficacy of returning to work was measured by the Return-to-Work Self-Efficacy Questionnaire (Chinese version) [26]. The Cronbach’s α coefficients ranged from 0.90 to 0.96. It contained 11 items, including reverse-scored items 2 and 6. The Likert scale was used to score the items. 1 point for ‘completely disagree’ and 6 points for ‘completely agree’. A score greater than 4.5 indicates an increased level of self-efficacy in returning to work, as determined by the average score of 11 items on the Return-to-Work Self-Efficacy Questionnaire.

The Connor-Davidson Resilience Scale [27] measured cancer patients’ mental resilience. Chinese scholars adjusted it into three dimensions, with a total of 25 items: tenacity (13 items), strength (8 items), and optimism (4 items). A Cronbach’s α coefficient of 0.89 was obtained, and a retest reliability of 0.87 was obtained. A Likert-5 scale is used, with 0 to 4 points for “never”, “rarely”, “sometimes”, “often” and “always”. The higher the total score, the better the mental resilience of patients.

Assessment of quality of life in cancer patients using the European Organization for Research and Treatment of Cancer Quality of Life Questionnaire-Core 30 (Chinese version) [28]. With 30 items, 15 dimensions could be distinguished: five functional dimensions (physical, role, cognitive, emotional, and social function), three symptom dimensions (fatigue, pain, nausea and vomiting), six single dimensions (shortness of breath, insomnia, loss of appetite, constipation, diarrhea, and financial difficulties), and one general health dimension. Among them, entries 29 and 30 are rated in 7 levels, from ‘very poor’ to ‘very good’ with 1–7 points respectively; the rest of the entries are rated in 4 levels: ‘no’, ‘somewhat’, ‘fairly’, and ‘very much’ are scored from 1 to 4, respectively. The reliability, validity, and reactivity of this Chinese scale have been proven. Higher scores on the functional dimension and general health status are associated with better quality of life for cancer patients, while higher scores on the symptom dimension are associated with worse quality of life for cancer patients.

A person’s competence at work was assessed using the Chinese version of the Work Ability Index questionnaire [29]. As part of the assessment, patients were asked to rate their physical and mental demands on a range of positions, their health, and their mental resources. The questionnaire consists of 7 dimensions, 13 questions, and a total score of 49. Based on the score, the work ability can be categorized as poor (7-27 points), moderate (28-36 points), good (37-43 points), and excellent (44-49 points). The Cronbach’s αcoefficients for all measures were above 0.70, which was an acceptable level of reliability. The higher the score, the more competent the patient is.

### Sample size

At the feasibility study stage, we applied ITT analysis to calculate the mean and standard deviation of the scores on the primary effectiveness outcome (adaptability to return to work) for the two groups of patients. As a result of the intervention, the control group’s return-to-work adaptability score was 85.730, with a standard deviation of 7.630. In the intervention group, the score was 92.730, with a standard deviation of 10.457. Using the sample size calculation method of ‘Tests for Two Means Differences’ (*N* = 2{(μ_α_ + μ_β_)σ/δ})^2^) [30, 31] and based on the above data, the power value was set to 0.90 and the alpha value was set to 0.05 (bilaterally) by using PASS 15.0 software in the ratio of 1:1. Ultimately, we needed 76 participants (38 in each group) to obtain statistical significance. In addition, consider that there was approximately 20% attrition during the intervention. Therefore, 96 participants (48 per group) were needed.

### Randomization and blinding

A randomization process was used to assign participants at a 1:1 ratio to either the intervention or control group after completing the baseline assessment. This study used Microsoft Excel’s ‘RANDBETWEEN’ function to generate a random number group using numbers numbered by an undergraduate medical student. In order to determine the intervention group, we sorted the numbers based on their size. The first half of the numbers were designated ‘1’. In the control group, the latter half of the numbers were coded as ‘2’. The numbers were recorded on sticky paper, sealed in opaque envelopes, and placed sequentially.

It is unlikely that the two groups could be blinded, as most patients themselves were aware that they were explicitly assigned to either the intervention or the control group. In accordance with the randomization procedure described above, the researchers who recruited the patients, collected the data and analyzed the data were blinded to prevent measurement bias. And they were not involved in grouping the patients or in the intervention. To avoid contamination effects, the control group was located on the chemotherapy I ward and the intervention group on the chemotherapy II ward. And no cases were dislodged in either group.

### Ethical considerations

This study was approved by the Ethics Committee of Nantong University (approval number: (2019)15) (February 15, 2019). Before the intervention, the researcher explained to the patients the purpose and content of the study, and informed them that they could withdraw from the study at any time.

### Data collection and analysis

Researchers who were not involved in patient recruitment and intervention entered and analyzed the data using SPSS 26.0. The level of statistical significance was set at α = 0.05. We analyzed the results of the Chi-square test or Fisher exact test by comparing the baseline demographics of patients in the intervention group to those in the control group. Use of the Kolmogorov-Smirnov test (K-S test) to assess the normality of the variable distributions. For normal distribution, use the mean and standard deviation; otherwise, the median and quartile were used. If normality and homogeneity of variance were satisfied, independent sample t test was used for comparison; otherwise, compare using the Wilcoxon signed rank test. To compare the overall changes of each outcome index between the intervention group and the control group at different measuring points, the main effects of the group effect, the time effect, and the group-by-time interaction effect were analyzed by establishing generalized estimation equations (GEE).

Moreover, the intervention was evaluated using per-protocol analysis (PP) and intention-to-treat analysis (ITT) due to the possibility of patients being excluded from the study and dropping out. In case of missing values during data analysis, the data from the previous measurement were shifted backwards to the data of the later measurement. Since the ITT analysis underestimates the intervention effect and the PP analysis overestimates the intervention effect, the credibility of the study is increased if these two analysis methods lead to essentially the same result [32]. Therefore, the effects of using both methods were assessed in this intervention study.

## Intervention

### Theoretical framework

**Fig. 1:**
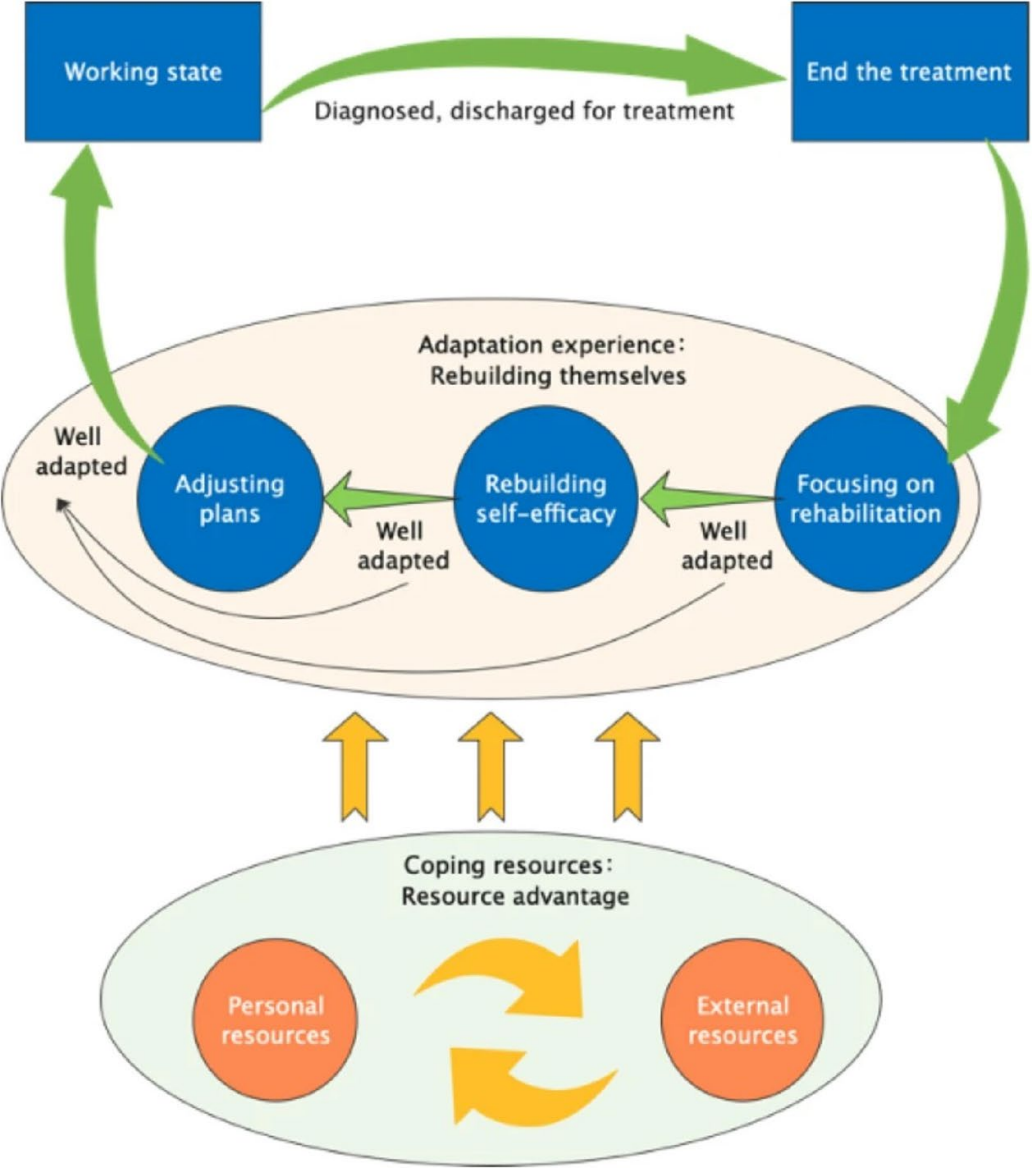
Cancer patients’ return-to-work adaptation experience and coping resource

At the beginning of the study, our team interviewed 30 cancer patients who had returned to work using a grounded theory approach and successfully created a model of ‘Adaptation Experience and Coping Resource Model for Cancer Patients to Return to Work’ [20] (Fig. 1). The model states that the adjustment experience of returning to work for cancer patients is a process of rebuilding through the utilization of superior resources. The adaptive experience consists of three themes: focus on rehabilitation, rebuild self-efficacy, adjust and plan. Focusing on rehabilitation runs through the whole adaptation experience of cancer patients; rebuilding self-efficacy is the key to adapting to their return to work; and adjusting and planning is the guarantee that they will adapt to their return to work. Based on this theoretical model, we conducted literature review and group discussion, structured interviews and Delphi expert consultation, and finally constructed a ‘Rebuilding Myself’ adaptive intervention protocol for cancer patients to return to work.

### Set up a research group

Before the intervention, the researcher set up a research group, which was mainly composed of 8 members, including a nursing professor who was engaged in the research of tumor psychosocial rehabilitation, and was responsible for team training, coordination and distribution, and quality control. 1 psychological counselor, who provided psychological counseling to patients when necessary. 4 graduate nursing students were responsible for collecting and analyzing data, recruiting patients and implementing intervention. Another 2 undergraduates majoring in medicine, were responsible for generating distribution sequences and analyzing data respectively.

### Implementation

If the eligible patients agreed to participate in the study, the researcher instructed them to read carefully and sign the informed consent form. Besides, the researcher also instructed patients to fill in the general situation questionnaire and baseline assessment questionnaire truthfully. A random distribution ratio of 1:1 was used to assign the patients to the intervention or control groups after completing the questionnaire. Using the patient’s electronic medical records and communicating with the medical staff in charge of the patient’s diagnosis and treatment, the researcher assessed the physical and psychological health status of cancer patients before intervention.

1. Control group: patients in the control group only received usual care, that is, researchers provided personalized medication guidance, follow-up visits and other information through WeChat or telephone. Meanwhile, the intervention implementer patiently answered the questions related to rehabilitation raised by patients, so as to keep in touch with patients and establish a long-term trust relationship.
2. Intervention group: On the basis of routine care, the intervention was carried out according to the intervention program [23]: firstly, to improve patients’ health management ability. Distribute health education manuals, encourage patients to learn about cancer rehabilitation, incorporate medical staff’s suggestions, guide patients to reflect on health risk factors, explore solutions, assist them in formulating rehabilitation plans and supervise their implementation; second, rebuild patients’ self-efficacy. Encourage patients to speak out about returning to work. They should communicate its positive significance, set up appropriate goals and record them in a diary, and firmly believe in recovery. Find out the attitudes of the patient’s relatives, colleagues, leaders, doctors and nurses towards his/her illness and return to work. Seek support from all parties for the patient. Ask peers to share their experience of returning to work and the difficulties they have overcome, so that they can act as role models. Encourage patients to adjust to their work routine, gradually complete their work tasks, and restore their confidence. Third, invite healthcare professionals to formulate a gradual return-to-work plan and career planning based on the patient’s recovery situation, as well as the actual situation of his/her family and unit, to maintain a balance between health and work, and encourage patients to actively seek support from their families and unit leaders (See Table 1). The intervention team determined the intensity of the intervention according to the patients’ recovery and needs. The total duration of the intervention was 3 months. The intervention was in the form of a combination of face-to-face (offline) and online (WeChat), and the intervention methods included communication and interviews, family meetings, diary writing, and mini-classes, etc. (The specific implementation requirements of the intervention protocol are shown in Table 2).

**Table 1:** The detailed information of the intervention protocol

Themes	Objectives	Contents	Methods
Focus on rehabilitation	1. Understand the importance of returning to work.	1. Encourage patients to express their views on returning to work, communicate with patients about the positive significance of returning to work, and help them build their belief in comprehensive recovery.	①
	2. Master the knowledge of physical and mental rehabilitation and self-management methods.	2. Carry out health education actively, and give out health education leaflets. Encourage patients to strengthen their study, master the knowledge of cancer recovery and keep a good attitude.	
		3. Guide patients to reflect on factors that are detrimental to their physical and mental recovery (such as bad living habits, environmental factors, personality defects, et al.), discuss targeted solutions with patients, and seek support from peers, family members, and medical staff when necessary.	
	3. Implement the self-management plan.	4. Ask patients’ healthcare providers about their health status and help them develop a self-health management plan.	
		5. Sign rehabilitation contracts with patients to enhance their compliance with health management.	
Rebuild self-efficacy	Be familiar with ways to improve self-efficacy.	1. Understand the views of the patient’s family members, peers, leaders, colleagues, and medical staff on patients’ illness and their return to work, and help them establish a correct view of rehabilitation.	
		2. Inform the patient’s family members, peers, leaders, colleagues, and medical staff of the importance of their care and support for their returning to work and complete recovery, and encourage them to offer their support.	
		3. Understand the condition of patients’ discussions with their family members, peers, colleagues, leaders, and medical staff about returning to work, ask them about their concerns and confusion on returning to work, and discuss solutions with patients.	②
		4. Encourage patients to perceive the support from their own beliefs, family members, leaders, colleagues, peers, medical workers, and other aspects, record it in the diaries and review it regularly to constantly firm their belief of comprehensive recovery.	①③
		5. Guide patients to find examples of ‘role models’ who have successfully returned to work after cancer, and share the experience and positive energy gained.	④
		6. Encourage patients to share their experiences of overcoming difficulties and achieving success in the past and the insights gained from them.	
		7. Self-confidence training:	
		① Positive psychological suggestion training: urge patients to smile to themselves every day, repeat positive words, and give patients timely affirmation and praise.	⑤
		② Mental resilience training: teach patients common stress coping skills, encourage patients to face pressure actively, and guide them to solve problems by clarifying and understanding issues, breaking complex problems into small steps, proposing solutions, and summarizing issues.	
		③ Patients are encouraged to gradually adjust their daily work and rest, and gradually integrate with the daily work and rest of the working stage.	①
		④ Encourage the patient to do things related to work gradually.	
Adjust and plan	Achieve the goal of returning to work gradually.	1. Invite medical staff to make scientific decisions on the appropriate time, position, and workload for patients to return to work according to their conditions.	
		2. Based on the advice of the medical staffs, ask patients about communication with their family members, peers, leaders, colleagues, etc., and urge the patient to actively seek support for returning to work if necessary.	
		3. Ask the patient about his/her work goal, discuss with him/her appropriate career goals according to his/her recovery condition, make a gradual career plan, and evaluate the relationship between health and work.	
		4. Summarize the contents of this intervention protocol to enhance the adaptability of returning to work.	

Methods: ①Individual communication and interview; ②Family meetings; ③Write diaries; ④Thematical communication; ⑤Mini-classes

**Table 2:** Specific implementation requirements for intervention protocol

Items	Contents
**Intervention methods**	Individual intervention through communicative interviews, family meetings, diary writing, and group intervention with mini-classes.
**Forms of intervention**	Form of a combination of face-to-face (one-on-one) and online (WeChat). Interventionist and patient discussed and chose a quiet, conversation-friendly location for the intervention.
**Intensity and duration of intervention**	Individualized intervention intensity and duration based on the patient’s own situation and needs, with a total intervention duration of 3 months.
**Intervention evaluation time**	Pre-intervention (at baseline), mid-intervention (at 6 weeks of intervention), post-intervention (at 12 weeks of intervention).
**Intervention evaluation indicators**	①Assessment Scale for Cancer Patients’ Adaptability to Return to Work ②Return-to-Work Self-Efficacy Questionnaire (RTW-SE) ③The Connor-Davidson Resilience Scale (CD-RISC) ④European Organization for Research and Treatment of Cancer Quality of Life Questionnaire-Core 30 (EORTC QLQ-C30) ⑤Work Ability Index questionnaire (WAI)

## Results

### Comparison of baseline data

#### Comparison of general conditions and effectiveness evaluation index scores between the two groups

The eligibility of 244 patients was assessed. The inclusion criteria were not met by 134 participants, while 14 declined to take part. Finally, 96 eligible participants were successfully recruited from January to September 2022. The intervention group consisted of 48 participants, and the control group consisted of the rest. At the 6th of the intervention, one patient in the intervention group and one patient in the control group discontinued the study due to lack of interest. After 12th week of intervention, 3 patients in the control group and 2 patients in the intervention group discontinued the study due to disinterest. One patient in each group was excluded because of disease metastasis and deterioration. Chi-square test or Fisher exact test was conducted to analyze the general conditions of the two groups of patients, and the results showed that the *P* values were all greater than 0.05, which was comparable (Table 3). The baseline analysis of the effectiveness evaluation index scores of the two groups showed that the *P* values were both greater than 0.05, which was comparable (Table 4).

**Table 3:** Comparison of the general conditions of the two groups of patients

Themes		Control group(*n* = 48)	Intervention group(*n* = 48)	χ^2^	*P*
Age	≤40	10	13	0.948	0.623
	41 ~ 50	21	22		
	> 50	17	13		
Gender	Male	12	8	1.011	0.315
	Female	36	40		
Marital Status	Married	47	47	0.000	1.000
	Divorced	1	1		
Religious beliefs	Yes	0	2	2.043	0.153
	No	48	46		
Educational Level	Primary education or no diploma	7	1	5.297	0.258
	Junior high school	20	24		
	High school or technical secondary school	11	13		
	Junior college	2	3		
	Bachelor degree or above	8	7		
Place of Residence	Countryside	13	15	0.731	0.694
	Town	16	18		
	City	19	15		
Medical Insurance	New Rural Medical Insurance	7	10	1.435	0.488
	Urban Resident Medical Insurance	11	7		
	Employee Medical Insurance	30	31		
Cancer type	Pericardial malignancy	0	1	14.605^a^	0.201
	Ovarian cancer	0	2		
	Lung cancer	5	1		
	Cervical cancer	1	1		
	Lymphoma	10	5		
	Breast cancer	27	29		
	Stomach cancer	3	2		
	Rectal cancer	1	1		
	Uterine cancer	1	0		
	Liver cancer	0	1		
	Malignant mole	0	1		
	Colon cancer	0	4		
Type of Employment	Worker	14	8	4.602	0.466
	Staff	10	12		
	Professional skill worker	7	6		
	Business and service personnel	2	6		
	Government agency personnel	2	4		
	Other	13	12		
Disease Stage	I	5	9	4.756	0.191
	II	30	33		
	III	11	6		
	IV	2	0		
Treatments	Radiation, chemotherapy, targeted therapy, or immunotherapy only	10	6	1.200	0.273
	Combining multiple treatment modalities	38	42		

a Fisher exact test

**Table 4:** Comparison of effectiveness evaluation index scores before intervention (baseline) between the two groups

Evaluation index		Control group	Intervention group	Statistics	*P*
**Adaptability to return to work (**x̄**±s)**		80.46 ± 8.73	80.83 ± 8.26	0.216	0.829
**Return to work self-efficacy (**x̄**±s)**		3.92 ± 0.41	3.93 ± 0.21	0.176	0.860
**Mental resilience (**x̄**±s)**		64.63 ± 12.24	64.69 ± 9.82	0.028	0.978
**Ability to work (**x̄**±s)**		27.93 ± 5.58	28.01 ± 4.55	0.080	0.936
	**Bodily function**	86.67(80.00, 93.33)	86.67(80.00, 93.33)	−0.224	0.823
**Quality of Life [M(P**_**25**_**，P**_**75**_**)]**	**Role function**	83.33(66.67, 83.33)	75.00(66.67, 83.33)	−0.565	0.572
	**Emotional function**	83.33(66.67, 97.92)	83.33(66.67, 97.92)	−0.127	0.899
	**Cognitive function**	100.00(83.33, 100.00)	100.00(83.33, 100.00)	−0.199	0.842
	**Social function**	75.00(66.67, 83.33)	66.67(66.67, 83.33)	−0.004	0.997
	**Fatigue**	77.78(66.67, 88.89)	66.67(66.67, 88.89)	−0.821	0.412
	**Pain**	100.00(83.33, 100.00)	100.00(83.33, 100.00)	− 0.877	0.380
	**Shortness of breath**	100.00(75.00, 100.00)	100.00(100.00, 100.00)	−0.611	0.541
	**Insomnia**	66.67(66.67, 100.00)	100.00(66.67, 100.00)	−0.579	0.563
	**Loss of appetite**	100.00(75.00, 100.00)	100.00(66.67, 100.00)	−1.829	0.067
	**Feel sick and vomit**	100.00(100.00, 100.00)	100.00(83.33, 100.00)	−1.450	0.147
	**Constipate**	100.00(100.00, 100.00)	100.00(66.67, 100.00)	−1.430	0.153
	**Diarrhea**	100.00(100.00, 100.00)	100.00(100.00, 100.00)	−0.297	0.767
	**Economic difficulties**	66.67(66.67, 100.00)	100.00(66.67, 100.00)	−0.692	0.489
	**General health**	50.00(43.75, 58.33)	58.33(50.00, 58.33)	−0.804	0.422

#### Comparison of effectiveness evaluation index scores between the two groups in the middle period (6th week) and late (12th week)

The results showed that in the middle period of the intervention, that is, the 6th week of intervention, the results of Per-Protocol analysis and Intention-To-Treat analysis showed that the scores of the two groups in the adaptability of returning to work, returning to work self-efficacy, mental resilience, and the dimension of cognitive function, general health status of quality of life were statistically significant (*P* < 0.05), while the scores of other indicators and dimensions were not statistically significant (*P* > 0.05) (Tables 5 and 6). After the intervention, that is, the 12th week of the intervention, the results of Per-Protocol analysis and Intention-To-Treat analysis showed that there were significant differences in the scores of the two groups in the adaptability of returning to work, returning to work self-efficacy, mental resilience, work ability, and physical function, emotional function, cognitive function, fatigue, insomnia and general health status of quality of life (*P* < 0.05), while there were no significant differences in the scores of other indicators and dimensions (Tables 7 and 8).

**Table 5:** Comparison of effectiveness evaluation index scores between the two groups at mid-intervention (at 6 weeks of intervention) (PP analysis)

Evaluation index		Control group	Intervention group	Statistics	*P*
**Adaptability to return to work (** $\overline{\boldsymbol{x}}\pm \textbf{s}$**)**		80.47 ± 8.64	85.91 ± 7.90	3.190	0.002^**^
**Return to work self-efficacy (** $\overline{\boldsymbol{x}}\pm \textbf{s}$**)**		3.93 ± 0.40	4.10 ± 0.30	2.331	0.022^*^
**Mental resilience(** $\overline{\boldsymbol{x}}\pm \textbf{s}$**)**		64.45 ± 12.24	69.17 ± 9.26	2.110	0.038^*^
**Ability to work (** $\overline{\boldsymbol{x}}\pm \textbf{s}$**)**		27.84 ± 5.45	29.82 ± 4.43	1.932	0.057
**Quality of Life [M(P**_**25**_**，P**_**75**_**)]**	**Bodily function**	86.67(80.00, 93.33)	86.67(80.00, 93.33)	−1.044	0.297
	**Role function**	83.33(66.67, 83.33)	83.33(66.67, 83.33)	−0.169	0.866
	**Emotional function**	83.33(66.67, 91.67)	83.33(75.00, 100.00)	−1.344	0.179
	**Cognitive function**	83.33(66.67, 100.00)	100.00(83.33, 100.00)	−2.660	0.008^**^
	**Social function**	83.33(66.67, 83.33)	83.33(66.67, 83.33)	−0.672	0.502
	**Fatigue**	77.78(66.67, 88.89)	77.78(66.67, 88.89)	−0.404	0.686
	**Pain**	100.00(83.33, 100.00)	100.00(83.33, 100.00)	−0.407	0.684
	**Shortness of breath**	100.00(100.00, 100.00)	100.00(100.00, 100.00)	−0.178	0.858
	**Insomnia**	66.67(66.67, 100.00)	100.00(66.67, 100.00)	−1.282	0.200
	**Loss of appetite**	100.00(66.67, 100.00)	100.00(66.67, 100.00)	−1.436	0.151
	**Feel sick and vomit**	100.00(100.00, 100.00)	100.00(83.33, 100.00)	−1.082	0.279
	**Constipate**	100.00(100.00, 100.00)	100.00(66.67, 100.00)	−1.211	0.226
	**Diarrhea**	100.00(100.00, 100.00)	100.00(100.00, 100.00)	−0.615	0.539
	**Economic difficulties**	66.67(66.67, 100.00)	100.00(66.67, 100.00)	−0.454	0.650
	**General health**	50.00(50.00, 58.33)	58.33(50.00, 66.67)	−3.294	0.001^**^

PS: ^*^P < 0.05, ^**^P < 0.01, ^***^P < 0.001

**Table 6:** Comparison of effectiveness evaluation index scores between the two groups in the mid-intervention period (at 6 weeks of intervention) (ITT analysis)

Evaluation index		Control group	Intervention group	Statistics	*P*
**Adaptability to return to work (** $\overline{\boldsymbol{x}}\pm \textbf{s}$**)**		80.52 ± 8.56	85.48 ± 8.37	2.869	0.005^**^
**Return to work self-efficacy (** $\overline{\boldsymbol{x}}\pm \textbf{s}$**)**		3.93 ± 0.40	4.10 ± 0.30	2.410	0.018^*^
**Mental resilience(** $\overline{\boldsymbol{x}}\pm \textbf{s}$**)**		64.71 ± 12.25	69.10 ± 9.17	1.991	0.049^*^
**Ability to work (** $\overline{\boldsymbol{x}}\pm \textbf{s}$**)**		28.05 ± 5.58	29.89 ± 4.41	1.785	0.078
**Quality of Life[M(P**_**25**_**，P**_**75**_**)]**	**Bodily function**	86.67(80.00, 93.33)	86.67(80.00, 93.33)	−1.195	0.232
	**Role function**	83.33(66.67, 83.33)	83.33(66.67, 83.33)	−0.092	0.927
	**Emotional function**	83.33(66.67, 91.67)	83.33(75.00, 97.92)	−1.153	0.249
	**Cognitive function**	83.33(66.67, 100.00)	100.00(83.33, 100.00)	−2.628	0.009^**^
	**Social function**	75.00(66.67, 83.33)	83.33(66.67, 83.33)	−0.657	0.511
	**Fatigue**	77.78(66.67, 88.89)	77.78(66.67, 88.89)	−0.294	0.769
	**Pain**	100.00(83.33, 100.00)	100.00(83.33, 100.00)	−0.159	0.873
	**Shortness of breath**	100.00(100.00, 100.00)	100.00(100.00, 100.00)	−0.409	0.682
	**Insomnia**	66.67(66.67, 100.00)	100.00(66.67, 100.00)	−1.544	0.122
	**Loss of appetite**	100.00(75.00, 100.00)	100.00(66.67, 100.00)	−1.629	0.103
	**Feel sick and vomit**	100.00(100.00, 100.00)	100.00(83.33, 100.00)	−1.321	0.187
	**Constipate**	100.00(100.00, 100.00)	100.00(75.00, 100.00)	−1.209	0.227
	**Diarrhea**	100.00(100.00, 100.00)	100.00(100.00, 100.00)	−0.614	0.539
	**Economic difficulties**	66.67(66.67, 100.00)	100.00(66.67, 100.00)	−0.692	0.489
	**General health**	50.00(50.00, 58.33)	58.33(50.00, 66.67)	−3.296	0.001^**^

PS:^*^P < 0.05, ^**^P < 0.01, ^***^P < 0.001

**Table 7:** Comparison of effectiveness evaluation index scores between the two groups in the later stage of intervention (after 12 weeks of intervention) (PP analysis)

Evaluation index		Control group	Intervention group	Statistics	*P*
**Adaptability to return to work (** $\overline{\boldsymbol{x}}\pm \textbf{s}$**)**		80.53 ± 8.33	90.46 ± 6.35	6.381	0.001^**^
**Return to work self-efficacy(** $\overline{\boldsymbol{x}}\pm \textbf{s}$**)**		3.92 ± 0.41	4.19 ± 0.33	3.427	0.001^**^
**Mental resilience(** $\overline{\boldsymbol{x}}\pm \textbf{s}$**)**		64.40 ± 12.25	72.50 ± 9.69	3.494	0.001^**^
**Ability to work (** $\overline{\boldsymbol{x}}\pm \textbf{s}$**)**		27.70 ± 5.16	30.58 ± 4.29	2.889	0.005^**^
**Quality of Life[M(P**_**25**_**，P**_**75**_**)]**	**Bodily function**	86.67(80.00, 93.33)	86.67(86.67, 93.33)	−1.996	0.046^*^
	**Role function**	83.33(66.67, 83.33)	83.33(66.67, 83.33)	−0.487	0.626
	**Emotional function**	75.00(66.67, 91.67)	91.67(81.25, 100.00)	−2.435	0.015^*^
	**Cognitive function**	83.33(66.67, 100.00)	100.00(83.33, 100.00)	−2.934	0.003^**^
	**Social function**	83.33(66.67, 83.33)	83.33(66.67, 83.33)	−1.212	0.226
	**Fatigue**	77.78(66.67, 88.89)	83.33(77.78, 88.89)	−2.531	0.011^*^
	**Pain**	100.00(83.33, 100.00)	100.00(83.33, 100.00)	−0.577	0.564
	**Shortness of breath**	100.00(100.00, 100.00)	100.00(100.00, 100.00)	−0.241	0.809
	**Insomnia**	66.67(66.67, 100.00)	100.00(66.67, 100.00)	−2.372	0.018^*^
	**Loss of appetite**	100.00(66.67, 100.00)	100.00(66.67, 100.00)	−0.753	0.452
	**Feel sick and vomit**	100.00(100.00, 100.00)	100.00(83.33, 100.00)	−1.096	0.273
	**Constipate**	100.00(100.00, 100.00)	100.00(91.67, 100.00)	−0.931	0.352
	**Diarrhea**	100.00(100.00, 100.00)	100.00(100.00, 100.00)	−0.037	0.971
	**Economic difficulties**	66.67(66.67, 100.00)	83.33(66.67, 100.00)	−0.312	0.755
	**General health**	50.00(50.00, 58.33)	66.67(56.25, 68.75)	−4.023	0.000^***^

PS:^*^P < 0.05, ^**^P < 0.01, ^***^P < 0.001

**Table 8:** Comparison of effectiveness evaluation index scores between the two groups in the later stage of intervention (at 12 weeks of intervention) (ITT analysis)

Evaluation index		Control group	Intervention group	Statistics	*P*
**Adaptability to return to work (** $\overline{\boldsymbol{x}}\pm \textbf{s}$**)**		80.79 ± 8.21	89.88 ± 7.22	5.756	0.000^***^
**Return to work self-efficacy (** $\overline{\boldsymbol{x}}\pm \textbf{s}$**)**		3.93 ± 0.40	4.19 ± 0.32	3.428	0.001^**^
**Mental resilience(** $\overline{\boldsymbol{x}}\pm \textbf{s}$**)**		64.77 ± 12.14	72.29 ± 9.54	3.375	0.001^**^
**Ability to work (** $\overline{\boldsymbol{x}}\pm \textbf{s}$**)**		27.97 ± 5.28	30.57 ± 4.23	2.668	0.009^**^
**Quality of Life[M(P**_**25**_**，P**_**75**_**)]**	**Bodily function**	86.67(80.00, 93.33)	86.67(86.67, 93.33)	−2.359	0.018^*^
	**Role function**	83.33(66.67, 83.33)	83.33(66.67, 83.33)	−0.481	0.631
	**Emotional function**	83.33(66.67, 91.67)	91.67(77.08, 100.00)	−2.021	0.043^*^
	**Cognitive function**	83.33(66.67, 100.00)	100.00(83.33, 100.00)	−2.732	0.006^**^
	**Social function**	75.00(66.67, 83.33)	83.33(66.67, 83.33)	−1.350	0.177
	**Fatigue**	77.78(66.67, 88.89)	77.78(77.78, 88.89)	−2.436	0.015^*^
	**Pain**	100.00(83.33, 100.00)	100.00(83.33, 100.00)	−0.485	0.628
	**Shortness of breath**	100.00(100.00, 100.00)	100.00(100.00, 100.00)	−0.420	0.675
	**Insomnia**	66.67(66.67, 100.00)	100.00(66.67, 100.00)	−2.673	0.008^**^
	**Loss of appetite**	100.00(75.00, 100.00)	100.00(66.67, 100.00)	−1.025	0.305
	**Feel sick and vomit**	100.00(100.00, 100.00)	100.00(83.33, 100.00)	−1.150	0.250
	**Constipate**	100.00(100.00, 100.00)	100.00(100.00, 100.00)	−0.980	0.327
	**Diarrhea**	100.00(100.00, 100.00)	100.00(100.00, 100.00)	−0.319	0.750
	**Economic difficulties**	66.67(66.67, 100.00)	100.00(66.67, 100.00)	−0.692	0.489
	**General health**	54.17(50.00, 58.33)	66.67(52.08, 66.67)	−3.844	0.000^***^

PS:^*^P < 0.05, ^**^P < 0.01, ^***^P < 0.001

### Overall test between intervention and control groups for the outcomes in GEE analysis

The test results of the GEE model effects of each evaluation index in the two groups of patients before and after intervention are shown in Table 9. Before intervention, at the sixth and twelfth weeks of intervention, the group effect, time effect, and group by time interaction effect were statistically significant in the comparison of adaptability to return to work and return to work self-efficacy between the two groups (all *P* < 0.05). There were no statistically significant differences (*P* > 0.05) in the group effects of mental resilience, work ability, and quality of life between the two groups at each intervention time point, but there were significant time effect and group by time interaction effect (*P* < 0.05).

**Table 9:** Overall test between intervention and control groups for the outcomes in GEE analysis (*n* = 96)

Evaluation time	Evaluation index	Group effect	Time effect	Group*Time effect
At baseline, At 6 weeks, At 12 weeks		Wald χ^2^ (*P* value)		
	Adaptability to return to work	8.874 (***P*** = 0.003^**^)	308.733 (***P*** = 0.000^***^)	275.964 (***P*** = 0.000^***^)
	Return to work self-efficacy	5.044 (***P*** = 0.025^*^)	74.562 (***P*** < 0.001^***^)	60.039 (***P*** < 0.001^***^)
	Mental resilience	3.301 (***P*** = 0.069)	242.242 (***P*** = 0.000^***^)	224.357 (***P*** = 0.000^***^)
	Ability to work	0.000 (***P*** = 0.985)	41.635 (***P*** < 0.001^***^)	37.958 (***P*** < 0.001^***^)
	Quality of life	2.350 (***P*** = 0.125)	38.044 (***P*** < 0.001^***^)	32.662 (***P*** < 0.001^***^)

PS:^*^P < 0.05, ^**^P < 0.01, ^***^P < 0.001

## Discussion

### The effect of this intervention

This study followed the formulation and evaluation of a complex intervention protocol [25] that included four steps: formative research, feasibility study, pilot study, and randomized controlled trial. Based on the theoretical model of ‘cancer patients’ return-to-work adaptation experience and coping resources’ constructed by our team members [20], the assessment scale of cancer patients’ adaptability [22] to going back to work was developed. Our team explored influencing factors in cancer patients’ adaptations to returning to work. Accordingly, an intervention protocol entitled ‘Rebuilding Myself’ was developed in order to improve cancer patients’ adaptability to returning to work after treatment [23]. In the initial phase of this study, we conducted a literature review and a group discussion. We revised and demonstrated this protocol based on structural interviews with stakeholders and Delphi expert consultations, and further improved it by conducting a feasibility and pilot study. The aim of this study was to conduct a randomized controlled trial to determine the effectiveness of this intervention.

Several studies [33] suggested that cancer patients’ return to work is multifaceted and requires psychological, vocational, and physical interventions. As a result of a systematic Cochrane review [34], physical, psychological, occupational and multidisciplinary interventions to promote cancer patients’ return to work were classified. Each type was defined. In the study, patients received multidisciplinary interventions that included physical, psychological and occupational aspects according to the items in the three themes of the protocol, namely ‘focus on rehabilitation’, ‘rebuild self-efficacy’ and ‘adjust and plan’, so as to help cancer patients achieve comprehensive rehabilitation. According to the results of the time effect and interaction effect, this intervention can effectively improve the adaptability and self-efficacy of returning to work, mental resilience, work ability, as well as the quality of life of cancer patients over time.

It has been suggested by Zamanzadeh et al. [35] that physical and psychological health provide an objective basis for the return to work of cancer patients. Within the theme of ‘Focus on rehabilitation’, research on sharing health education knowledge with patients, encouraging patients to strengthen their rehabilitation knowledge, reflecting on factors that are not conducive to their physical and mental rehabilitation, and formulating and implementing plans to manage their own health can help patients alleviate symptoms of fatigue and insomnia and improve their physical and emotional function, thereby restoring their physical and mental health. The researchers encouraged patients to independently acquire relevant examples of ‘role models’ and draw indirect experiences and positive energy from them as part of the ‘rebuilding self-efficacy’ theme. Researchers also assist patients in feeling emotionally supported by their family, peers, colleagues, and medical staff, thereby changing their negative attitude towards returning to work, improving their cognitive function, and making patients think about returning to work and then improving their abilities at work. In addition, researchers encouraged the participants to share their previous successes in overcoming difficulties. They also help them gain direct experience through self-confidence training, which helps patients rebuild their self-efficacy. As Bandura et al. found, these results are consistent [36]. Within the theme of ‘Adjust and plan’, this intervention mobilized patients’ internal and external superordinate resources, and patients were guided to seek occupational support from family and society, adjust their work goals, develop career plans step by step, balance health and work, and then gradually achieve comprehensive recovery from their physical and mental illnesses. Through the interventions in these three themes, patients actively respond to various challenges in the process of returning to work (such as physical and mental trauma, low self-efficacy, maintaining the balance between health and work, etc.), strive to adapt themselves (such as introspection and adjustment, strengthening study, etc.), restore bio-psychological-mental stability, and finally improve their adaptability in returning to work.

However, there was no significant difference between the two groups when it came to pain, breathlessness, anorexia, nausea, vomiting, constipation, diarrhea, financial difficulties, social function, and role function before, during, and after the intervention. Possible reasons were as follows: symptoms of cancer patients such as pain, anorexia, nausea and vomiting, constipation, and diarrhea could be relieved in time through drug intervention. In addition, the treatment and rehabilitation of cancer patients was a long process, and the long-term side effects of cancer and its treatment could persist [37], while the intervention time of this study was only 3 months. Thus, the breathlessness, role, and social function of cancer patients were not significantly improved in a short period of time.

### Implications for nursing practice

Doctors, nurses, patients and their families, colleagues and other staff had to work together to implement this intervention. Researchers should communicate fully with patients, assess their physical and mental state, and establish a trusting relationship with them prior to the intervention. Researchers should fully consider the patients’ situation and needs and formulate personalized intervention content. Meanwhile, researchers should flexibly adjust the content of the intervention according to patients’ physical and mental health status. Furthermore, our research team found that the experience of cancer patients using superior resources to rebuild themselves and adapt to return to work includes three dimensions that run through the whole rehabilitation process. Therefore, researchers should simultaneously promote the content of the three themes as part of the intervention to ensure comprehensive rehabilitation of patients.

### Strengths and limitations

This study focused for the first time on the adaptability of cancer patients returning to work and conducted a randomized controlled trial to verify the effectiveness of the intervention protocol, with the aim of improving the adaptive capacity of cancer patients returning to work, promoting their reintegration into society and supporting their physical, mental and social rehabilitation. Secondly, the four steps of the methodology ‘Formulation and evaluation of a complex intervention plan’ were implemented in this study. Based on the ‘Adaptation Experience and Coping Resource Model for cancer patients to return to work’, we created the ‘Rebuilding Myself’ intervention protocol for the first time. Finally, the team will continue to conduct large-sample randomized controlled trials and qualitative studies in the future in order to gain a deeper understanding of cancer patients’ return-to-work experiences.

There were several limitations of the study that should be taken into account. Firstly, due to the limited time frame, only the quantitative evaluation method was used in this study to examine the immediate effect of the intervention program before, during, and after the intervention, and participants may be followed up in the future to further examine the long-term effect of the intervention. Meanwhile, qualitative and quantitative evaluations, such as interviews, can be combined in the future to comprehensively analyze the effect of the intervention. Secondly, restricted by the epidemic situation and research conditions, the regional distribution of patients recruited in this study was still limited. So, in the future, we can select several hospitals and communities in other provinces in China to carry out multi-center randomized controlled trial research, so as to better help cancer patients improve their adaptability to returning to work and achieve comprehensive physical and mental recovery. Finally, due to the short follow-up period of this study and the fact that the cancer patients were in the recovery phase, we could not use ‘return to work’ as an outcome indicator. We will continue to monitor their return to work in subsequent studies.

## Conclusions

In this study, a randomized controlled trial was conducted on the basis of the intervention plan drawn up in the initial phase. It was found that ‘Rebuilding Myself’ intervention was effective in improving the adaptability and self-efficacy to return to work, mental resilience, work ability, and quality of life of cancer patients. In this study, patients received multidisciplinary interventions that included physical, psychological and vocational aspects according to the three themes of the intervention protocol. The ‘Focus on rehabilitation’ theme focused on promoting the physical and psychological recovery of cancer patients; ‘Rebuilding self-efficacy’ improved patients’ self-efficacy in returning to work by boosting their self-confidence; and ‘Adjust and plan’ guided patients to balance the relationship between health and work and gradually realize their comprehensive physical, psychological, and social rehabilitation.

## Data Availability

The data of individual participants behind the results reported in this paper will be shared with researchers who provide reasonable suggestions on methods after identification. The datasets analysed during the current study available from the corresponding author on reasonable request.
